# Supplementary material for: Perioperative patient safety management activities: A modified theory of planned behavior
Source: PLoS One. 2021 Jun 25;16(6):e0252648. doi: 10.1371/journal.pone.0252648 (PMC8232430; doi:10.1371/journal.pone.0252648)
Supplement: Attachment — Submitted filename: Reviewer Recommendation and Comments for Manuscript Number PONE.docx [file pone.0252648.s001.docx]

**Reviewer Recommendation and Comments for Manuscript Number PONE-D-20-31121**

The research question is interesting and cogent, and the research is based on a sound literature ground concerning safety behaviors. I think the research is worth publishing, but I address some aspects that could be clarified by the authors.

1. Line 83: the authors make reference to the safety management system. It si not clearly explained how these aspects could hinder safety. The authors do not explicitly refer to organizational culture factors, like the kind of leadership, the blame culture, the safety climate (which are implicit in the safety management tool used in the research).

2. Line 125: The authors should explain why they chose the Job Content  Questionnaire developed by Karasek et al.

and not other tools. What is the rationale of this choice?

3. Line 138: The 12-item scale is based on an unpublished study by Moon. The authors should provide evidence of the validity of the scale

4. Line 155. Also the safety management activity instrument, developed by Jeong, is an unpublished research and the authors should provide evidence of its validity

5. Line 279: The issue of behavioral control is controversial. The internal locus of control is generally considered to be a better predictor f safe performance, but in extreme situations it could also represent an excessive trust in one’s own skills and a deliberate exposure to reckless actions. The authors could provide a deeper explanation of these results.

Line 302: the authors mention among the limitations of the research the fact that it was based on self-report data. Self-report tools may be biased by social desirability, especially when they are related to errors, violations, and safety issues. The authors mention objective measurements such as observational surveys, however, I think it could have be useful to add other kind of objective data to the analysis, for instance concerning the rate of adverse events, injuries, near misses, etc.
